# Influence of Different Precursors on Properties and Photocatalytic Activity of g-C_3_N_4_ Synthesized via Thermal Polymerization

**DOI:** 10.3390/ma18112522

**Published:** 2025-05-27

**Authors:** Debora Briševac, Ivana Gabelica, Floren Radovanović-Perić, Kristina Tolić Čop, Gordana Matijašić, Davor Ljubas, Lidija Ćurković

**Affiliations:** 1 Faculty of Mechanical Engineering and Naval Architecture, University of Zagreb, Ivana Lučića 5, 10000 Zagreb, Croatia; ivana.gabelica@fsb.unizg.hr (I.G.); davor.ljubas@fsb.unizg.hr (D.L.); 2 Faculty of Chemical Engineering and Technology, University of Zagreb, Marulićev trg 19, 10000 Zagreb, Croatia; fradovano@fkit.unizg.hr (F.R.-P.); ktolic@fkit.unizg.hr (K.T.Č.); gmatijas@fkit.unizg.hr (G.M.)

**Keywords:** graphitic carbon nitride, thermal polymerization, characterization, photocatalysis, procaine

## Abstract

In this research, an emerging, non-metallic photocatalyst was prepared by the thermal polymerization method from three different precursors: urea, melamine, and three mixtures of melamine and cyanuric acid. Graphitic carbon nitride (g-C_3_N_4_) samples from urea and melamine were synthesized in a muffle furnace at three different temperatures: 450°, 500°, and 550 °C for 2 h, while the samples made of a mixture of melamine and cyanuric acid (with mass ratios of 1:1, 1:2, and 2:1) were synthesized at 550 °C for 2 h. All the samples were characterized in order to determine their chemical and physical properties, such as crystallite size and structure, and phase composition by the following techniques: Fourier transform infrared spectroscopy (FTIR), X-ray diffraction analysis (XRD), and scanning electron microscopy (SEM) with energy-dispersive X-ray spectroscopy (EDS). Nitrogen adsorption/desorption isotherms were used to investigate the Brunauer, Emmett, and Teller (BET) specific surface area and Barrett–Joyner–Halenda (BJH) pore size distribution. Band gap values were determined by diffuse reflectance spectroscopy (DRS). Furthermore, adsorption and photocatalytic degradation of the local anesthetic drug procaine were monitored under UV-A, visible, and simulated solar irradiation in a batch reactor. Kinetic parameters, as well as photocatalytic mechanisms using scavengers, were determined and analyzed. The results of the photocatalysis experiments were compared to the benchmark TiO_2_ Evonik Aeroxide P25. The results indicated that the g-C_3_N_4_ sample synthesized from urea at 500 °C for 2 h exhibited the highest degradation rate of procaine under visible light.

## 1. Introduction

Water quality is essential to both environmental and human health, and it is increasingly impaired by the many organic micropollutants (OMPs). OMPs are usually present in water at trace levels (usually ng L^−1^ to μg L^−1^) and include substances such as pharmaceuticals, personal care products, industrial chemicals, and pesticides. Unlike conventional pollutants, OMPs are often resistant to degradation and thus may persist in the environment, causing bioaccumulation and toxicity for aquatic ecosystems and even human health [1,2]. One of these OMPs is procaine (PRO), an active pharmaceutical ingredient used as a local anesthetic in medical and veterinary practices. PRO has been detected in both wastewater effluents and natural water bodies [3]. The continued persistence of procaine in water indicates the need for effective remediation technologies with the capability to remove trace organic pollutants from water.

Conventional water treatment methods, such as coagulation, sedimentation, and filtration, generally cannot remove the OMPs present in aqueous matrices, especially at trace-level concentrations. Therefore, advanced oxidation processes (AOPs) have gained attention as promising solutions for degrading resistant OMPs [4]. AOPs are based on the generation of highly reactive species, mainly hydroxyl radicals (^•^OH), which can non-selectively oxidize organic pollutants, breaking them down into smaller, less harmful compounds or mineralizing them entirely to carbon dioxide and water. One of the AOPs is photocatalysis, which uses light to activate a semiconductor catalyst, producing reactive oxygen species (ROS) and enabling the degradation of complex pollutants [5].

Among different kinds of photocatalysts, titanium dioxide (TiO_2_) is one of the most widely studied due to its advantages, including high oxidative power, excellent chemical stability, nontoxicity, and affordability. TiO_2_ can absorb UV light energy, which promotes the shift of electrons from the valence band (VB) to the conduction band (CB), leading to the generation of electron-hole pairs that can interact with water molecules to produce ROS. However, TiO_2_ has a significant application limitation due to its large band gap of approximately 3.2 eV, limiting its activation primarily to the UV spectrum, which makes up only about 5% of the solar spectrum. Therefore, TiO_2_-based photocatalytic processes are inefficient under visible light, limiting their applications for water treatment, especially in outdoor or solar-powered systems [6].

The disadvantages of TiO_2_ activation under visible light have driven extensive research into novel photocatalysts that can utilize visible light, which accounts for roughly 45% of the solar spectrum. One of the most promising emerging materials is graphitic carbon nitride (g-C_3_N_4_), a polymeric semiconductor mainly based on carbon and nitrogen atoms. Compared to TiO_2_, g-C_3_N_4_ has a relatively narrow band gap of about 2.7 eV, allowing visible light absorption and electron-hole generation under sunlight irradiation. That unique property makes g-C_3_N_4_ very attractive for photocatalytic applications due to the possibility of using solar energy in an efficient way in water treatment processes [7].

Graphitic carbon nitride has a layered structure similar to graphite, with a large surface area that promotes interaction with pollutants and thus enhances photocatalytic activity. Besides that, it also has an adjustable electronic structure, high chemical stability, and low production cost, making it an ideal candidate for many photocatalytic applications. It also has high stability in both acidic and basic environments, which increases its application in different fields, especially in environmental rehabilitation and water treatment processes [8]. G-C_3_N_4_ can be synthesized from easily available, low-priced nitrogen-rich precursors such as urea [9], thiourea [10], melamine [11], cyanamide [12], and dicyandiamide [13]. Properties of g-C_3_N_4_, such as its surface area, crystallinity, and band structure, can be modified depending on the precursor choice and synthesis method. Typical synthesis methods include thermal polymerization [14], electrodeposition [15], hydrothermal [16], solvothermal [17], and microwave-assisted methods [18].

The photocatalytic activity of g-C_3_N_4_ has enabled its application in a variety of environmental and energy applications. In water treatment, g-C_3_N_4_ has shown significant potential for the degradation of a wide range of pollutants, including dyes [19], pesticides [20], and pharmaceuticals [21] such as procaine. Under visible light irradiation, g-C_3_N_4_ generates electron-hole pairs that participate in the formation of ROS, effectively decomposing OMPs without forming secondary contaminants. Furthermore, its stability under visible light irradiation and a range of environmental impacts makes it appropriate for long-term applications, providing a green and cost-effective method for the elimination of organic pollutants. Besides pollutant degradation, g-C_3_N_4_ has also been investigated for photocatalytic hydrogen production [22], CO_2_ reduction [23], nitrogen fixation [24], heavy metals removal [25], and air purification [26], proving its potential beyond water treatment. Further investigation, e.g., [27], showed that an oxygen self-doping strategy can enable highly efficient visible-light-driven photocatalysis by tailoring the electronic structure of g-C_3_N_4_, offering a sustainable and metal-free approach for environmental remediation through markedly enhanced degradation of organic pollutants. Additionally, the Z-scheme Cs_3_BiBr_9_ nanoparticles@porous C_3_N_4_ tubular heterojunction offers a lead-free and sustainable photocatalytic platform with significantly enhanced solar-driven oxidation performance, for example, of benzylic alcohol, representing a promising route for the selective production of value-added organics at scale [28].

This research aims to prepare g-C_3_N_4_ nanoparticles via a thermal polymerization method using different precursors, including urea, melamine, and a combination of melamine and cyanuric acid at different temperatures. The systematic comparison of g-C_3_N_4_ photocatalysts derived from these different precursors offers detailed insight into how the precursor choice and synthesis temperature influence surface morphology, structure, and photocatalytic activity. A key novelty lies in identifying the specific optimal synthesis conditions (precursor and temperature) that yield the g-C_3_N_4_ material showing the highest procaine degradation rate, particularly highlighting superior performance under visible light irradiation. Thermal polymerization represents a cost-effective method for producing efficient photocatalysts. The photocatalytic efficiency for the degradation of procaine under various light sources in correlation with the properties of the produced g-C_3_N_4_ samples was investigated. Moreover, insight into the degradation mechanisms was obtained via scavenger testing.

## 2. Materials and Methods

### 2.1. Preparation of g-C_3_N_4_ Samples

Samples of graphitic carbon nitride nanoparticles were prepared by the thermal polymerization method in a muffle furnace (Inko LP-08, Zagreb, Croatia) from the following precursors: urea (NH_2_CONH_2_, 99.5%, VWR International, LLC, Radnor, PA, USA), melamine (C3H_6_N_6_, 99%, Thermo Scientific Chemicals, Waltham, MA, USA), and a mixture of melamine and cyanuric acid (C_3_H_3_N_3_O_3_, 99%, Thermo Scientific Chemicals, Waltham, MA, USA). The melamine and cyanuric acid mixture was prepared in the following mass ratios: 1:1, 1:2, and 2:1. The powder precursors were calcined in a muffle furnace in a covered ceramic crucible at different temperatures, as described in Table 1. The urea and melamine were prepared at three different temperatures: 450, 500, and 550 °C, while the mixtures of melamine and cyanuric acid were prepared at 550 °C only, according to the literature findings [29,30]. After heating, the resulting particles (Figure S1) were ground in a mortar and characterized using the methods described below.

### 2.2. Characterization of Prepared g-C_3_N_4_ Samples

A Fourier transform infrared spectroscopy (FTIR) analysis of the g-C_3_N_4_ samples was conducted using a Shimadzu IRSpirit spectrometer (Tokyo, Japan) equipped with an ATR (attenuated total reflectance) attachment in the wavenumber range from 400 to 4000 cm^−1^. X-ray diffraction (XRD) was used to identify the crystalline phases present in the samples. The XRD measurements were performed with a Bruker D8 Advance diffractometer (Billerica, MA, USA), using CuKα radiation at 40 kV and 25 mA. Data was collected in the Bragg–Brentano configuration, covering a 2θ range from 10 to 80 degrees (or 10 to 60 degrees), with a step size of 0.02°, and a step time of 0.6 s. Diffuse reflectance spectroscopy (DRS) was performed on a PerkinElmer Lambda 1050+ spectrophotometer (Shelton, CT, USA) for all the samples. The reflectance spectra were recorded in the UV-VIS region between 250 and 750 nm, with a resolution of 1 nm. Barium sulfate (BaSO_4_) served as the reference standard. The band gap energy (*E*_g_) was calculated from the reflectance spectra using the Kubelka–Munk function or Tauc plot. Nitrogen adsorption/desorption isotherms were measured using a Micromeritics ASAP-2000 instrument (Norcross, GA, USA) at −196 °C. Before the measurements, the samples were degassed at 150 °C until the vacuum reached below 50 mm Hg to eliminate the adsorbed impurities.

The specific surface area (*S*_BET_) was calculated using the BET model based on five points within the relative pressure range of 0 to 0.2. The pore volume (*V*_p_) and pore size distribution were determined from the nitrogen adsorption branch using the Barrett–Joyner–Halenda (BJH) method. SEM was conducted via SEM Tescan Vega III Easyprobe, with an accelerating voltage of 10 kV, equipped with secondary (SE) and backscattered electron (BSE) detectors (Tescan, Brno, Czech Republic).

### 2.3. Adsorption, Photolytic, and Photocatalytic Testing

The adsorption process of the photocatalyst and photocatalytic degradation of PRO were evaluated in a batch photoreactor. The adsorption was investigated in the dark at a constant stirring of 50 mg g-C_3_N_4_ samples dispersed in 100 mL PRO solution (10 mg L^−1^) for 2 h. Samples were collected from the batch photoreactor at specific intervals (0, 1, 5, 10, 20, 30, 45, 60, 90, and 120 min) and filtered through a 0.45 µm mixed cellulose ester membrane. The filtrates were analyzed using a UV-VIS spectrophotometer (HEWLETT PACKARD, Model HP 8430, Palo Alto, CA, USA) at 290 nm, which corresponds to the maximum absorption peak of PRO. All the g-C_3_N_4_ samples achieved equilibrium sorption within 30 min.

In the photocatalytic experiments, the same amounts of photocatalyst in the PRO solution were first stirred in the dark for 30 min to achieve adsorption/desorption equilibrium and later irradiated under visible (model OSRAM Endura Flood 840 GD, Ledvance GmbH, Osram, Munich, Germany; 400 and 600 nm, 100 W), UV-A (model UVAHAND LED, Dr. Hönle AG, UV-Technologie, Gilching, Germany; 365 nm, 70 W) and simulated solar lamps (model SOL500, Dr. Hönle AG, UV-Technologie, Gilching, Germany; 430 W). The distance between the complexes in a photoreactor and different types of lamps was determined to achieve the same amount of UV-A radiation, equaling 5 cm for a visible, 30 cm for a UV-A, and 22 cm for the simulated solar lamp. All the photocatalytic experiments were conducted for 2 h with constant stirring using a magnetic stirrer. Throughout the experiment, the temperature was maintained at 25 °C using a thermostatic bath (Lauda ECO Silver RE415, Lauda-Königshofen, Germany). Additionally, the photolytic activity of the PRO solution was tested by following the same procedure as the photocatalytic experiments, but without the addition of the g-C_3_N_4_ photocatalyst. All the experiments were conducted in ultrapure water. The results of the photocatalytic degradation of PRO using g-C_3_N_4_ were compared to a commercially available TiO_2_ photocatalyst (Aeroxide TiO_2_ P25, Evonik Industries, Essen, Germany). All the experiments were conducted in triplicate to ensure reproducibility, with variation across the three measurements remaining below 5% for all the samples.

### 2.4. Scavenger Test

The scavengers that were used in this study were isopropanol (Gram mol, Zagreb, Croatia), formic acid (Lach-Ner s.r.o., Neratovice, Czech Republic), p-benzoquinone (Merck KGaA, Darmstadt, Germany), and sodium azide (Kemika, Zagreb, Croatia) as a hydroxyl radical (^•^OH), hole (h^+^) scavenger, superoxide radical (O_2_^•−^), and singlet oxygen scavenger (^1^O_2_), respectively. The scavengers were added in a batch reactor at the beginning of the adsorption and photocatalytic tests in the molar ratio pollutant/scavenger 1:100, except for the p-benzoquinone, which was added in a ratio of 1:50 due to limitations during the analytical test.

The presence of radical species responsible for procaine degradation was monitored using an HPLC–DAD system (Agilent 1100, Santa Clara, CA, USA). A C18 XBridge column (150 mm × 4.6 mm, 3.5 μm) was used as a stationary phase from which the pharmaceutical was eluted by changing gradient of the mobile phases consisting of 0.1% formic acid in Milli-Q water (A) and 0.1% formic acid in acetonitrile (B) at a flow rate of 0.5 mL min^−1^. The gradient started with 95% of A and was maintained for 1 min. Within the next 5 min, the composition of mobile phase A decreased to 35%. After holding 35% of A for 6 min, at 12.01 min, the composition phase was returned to the initial conditions for column equilibration for 3 min. The injection volume was 30 µL. Procaine was detected at 294 nm and had a retention time of 8.1 min.

## 3. Results and Discussion

### 3.1. Results of Characterization of Prepared g-C_3_N_4_ Samples

**Figure 1:**
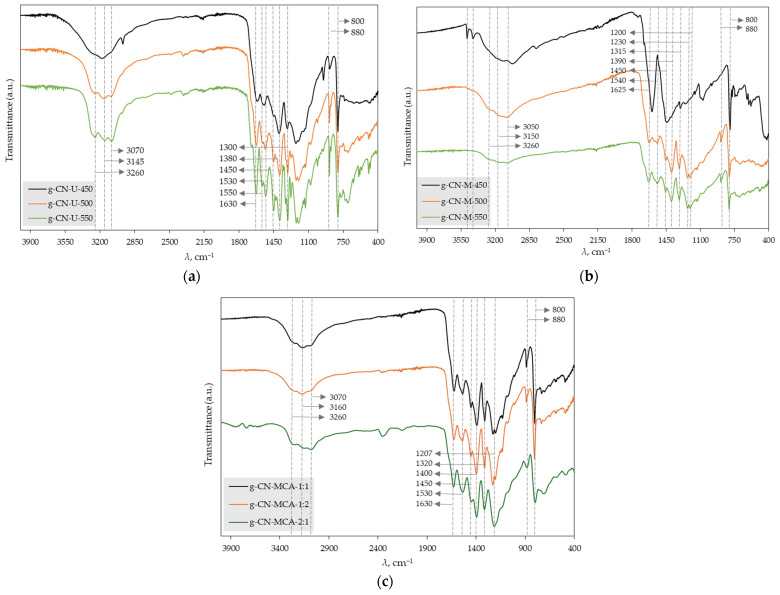
FTIR spectrum of g-C_3_N_4_ samples synthesized from (**a**) urea, (**b**) melamine, and (**c**) the complex of melamine and cyanuric acid.

Figure 1a presents the FTIR spectrum of g-C_3_N_4_ samples synthesized from urea at temperatures of 450, 500, and 550 °C. For the samples g-CN-U-500 and g-CN-U-550, characteristic vibrational bands of the triazine aromatic ring are visible around 800 and 880 cm^−1^. Similar findings were reported in a study on urea-based g-C_3_N_4_ nanoparticles calcinated at 350–650 °C, where *s*-triazine ring vibrations were observed at 815 cm^−1^ [31]. Additionally, in the region between 1200 and 1600 cm^−1^, vibrational stretches of C–N and C=N can be seen. These observations align with another study on tri-*s*-triazine g-C_3_N_4_ synthesized from urea at temperatures ranging from 400 to 600 °C, where similar C–N and C=N stretches were detected in the range of 1250 to 1650 cm^−1^ [32]. In the 3000 to 3300 cm^−1^ region, stretches corresponding to primary and secondary amines from the CN heterocycle are present, likely originating from the adsorbed water’s –OH group. These results are consistent with previous studies that detected N–H and O–H vibrational stretches in the 3000 to 3500 cm^−1^ range [33].

**Figure 2:**
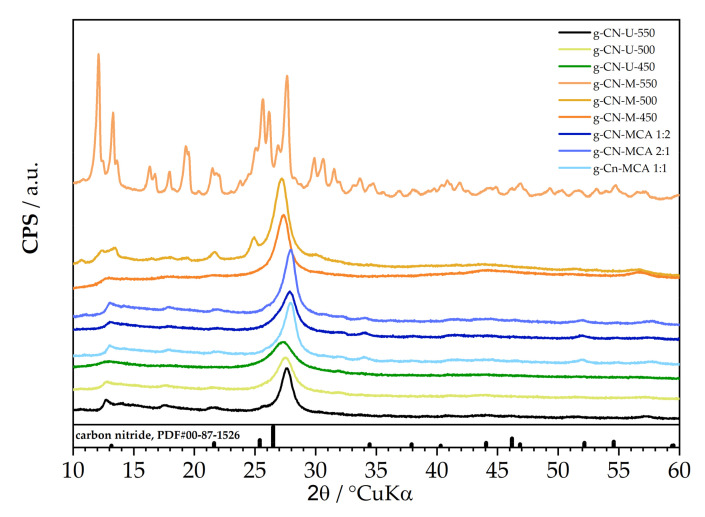
XRD diffractograms of g-C_3_N_4_ samples synthesized from urea, melamine, and a complex of melamine and cyanuric acid.

Figure 1b shows the FTIR spectrum of g-C_3_N_4_ synthesized from melamine at 450, 500, and 550 °C. Similar to the urea-derived samples, the *s*-triazine aromatic ring bands at around 800 and 880 cm^−1^ can be seen for the samples g-CN-M-500 and g-CN-M-550, along with C–N and C=N vibrational stretches in the 1200 to 1600 cm^−1^ region and amine stretches from the CN heterocycle between 3000 and 3300 cm^−1^. These findings match those of a study on g-C_3_N_4_ synthesized from melamine at temperatures of 450 to 650 °C, where *s*-triazine aromatic vibrations appeared at 812 cm^−1^, and C–N and C=N stretches were found in the 1241 to 1640 cm^−1^ range, with amine stretches between 3000 and 3600 cm^−1^ [32]. Additionally, for the sample g-CN-M-450, broader peaks occur between 1000 and 1800 cm^−1^, which means that the occurring products, such as ammelide, have not been fully transformed into g-C_3_N_4_. The same is observed in the XRD results in Figure 2 [34].

Figure 1c shows the FTIR spectrum of g-C_3_N_4_ synthesized from the complex of melamine and cyanuric acid in different mass ratios: 1:1, 1:2, and 2:1. As well as for the urea and melamine-derived samples, characteristic triazine bands are visible at around 800 and 880 cm^−1^ for all three mass ratios. Also, C-N and C=N vibrational stretching are visible in the 1200 to 1600 cm^−1^ region, and amine stretches from the CN heterocycle between 3000 and 3300 cm^−1^. The obtained g-C_3_N_4_ shows peaks at 570 and 690 cm^−1^, corresponding to melamine and cyanuric acid complex [35].

Figure 2 displays the diffractograms of g-C_3_N_4_ samples synthesized from urea, melamine, and a complex of melamine and cyanuric acid. The diffractograms of urea-synthesized samples show one prominent diffraction peak and several weaker intensity peaks. The peaks at 2θ = 13° and 2θ = 27.7° are characteristic of the g-C_3_N_4_ phase, according to ICDD PDF#00-87-1526. Similar results were found in the previously mentioned study, where XRD analysis revealed two peaks, one at 2θ = 13.1° and another at 2θ = 27.6°. As noted in the mentioned study, the peak at 2θ = 27.6°, indexed as the (002) plane, corresponds to the interplanar stacking of aromatic structures. In contrast, the smaller peak at 2θ = 13.1°, indexed as the (100) plane, relates to the in-plane structural packing motif [27]. Additionally, as the temperature increases from 450 °C to 550 °C, a shift in the peak from 27.3° to 27.7° is observed, confirming the formation of the g-C_3_N_4_ phase. By increasing the temperature, the dominant (002) peak becomes narrower and more intense, which indicates an increase in the crystallinity of the sample, while the change in the (100) peak indicates a higher degree of polymerization of g-C_3_N_4_. When the carbon nitride ICDD PDF#00-87-1526 pattern is included for comparison, the diffraction maxima for g-C_3_N_4_ shifts slightly to 27.7°.

In the diffractograms of g-C_3_N_4_ samples synthesized from melamine, the melem phase, which forms between 370 and 500 °C during the thermal processing of melamine, is indicated. The sample g-CN-M-450 consists solely of the melem phase, while increasing the temperature leads to the formation of the g-C_3_N_4_ phase [34]. Similar to the urea-derived samples, the carbon nitride ICDD PDF#00-87-1526 is used for comparison, although the positions of the diffraction peaks differ slightly. In the sample g-CN-M-550, a prominent diffraction peak, along with several weaker intensity peaks, is evident.

Melamine and cyanuric acid complex show fully formed, crystalline g-C_3_N_4_, evident by the largest 2θ shift towards higher values. No significant difference is observed for different melamine/cyanuric acid ratios.

**Figure 3:**
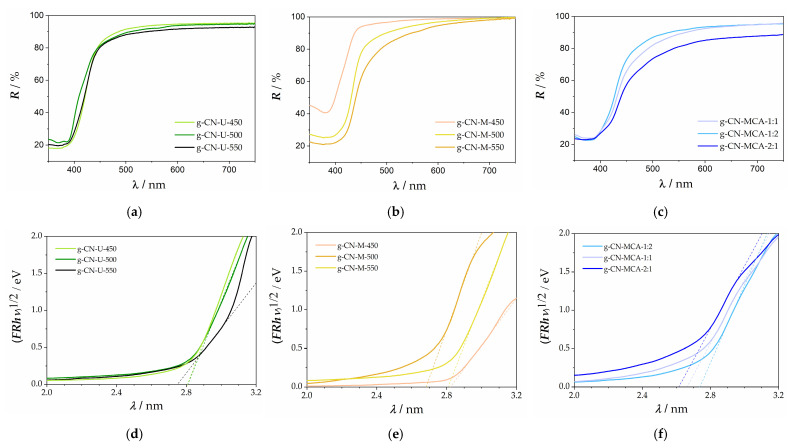
Reflectance spectra of g-C_3_N_4_ samples synthesized from (**a**) urea, (**b**) melamine, and (**c**) the complex of melamine and cyanuric acid. Tauc’s graphical representation of g-C_3_N_4_ samples synthesized from (**d**) urea, I melamine, and (**f**) the complex of melamine and cyanuric acid.

Figure 3a presents the reflectance measurements for g-C_3_N_4_ samples synthesized from urea at temperatures of 450, 500, and 550 °C. The graphs in Figure 3d show that as the processing temperature increases from 450 to 550 °C, the indirect band gap (*E*_g_) increases from 2.81 eV to 2.88 eV. The values listed in Table 2 are slightly higher than those reported in previous research [31]. The band gap variation can be correlated to the processing temperature by considering particle growth, which reduces the specific surface area and alters the defect structure of the material [36].

Figure 3b displays the reflectance spectra for g-C_3_N_4_ samples synthesized from melamine at 450, 500, and 550 °C. As the processing temperature increases from 450 to 550 °C, a redshift in the reflectance towards longer wavelengths (from about 400 to 420 nm) is observed. Correspondingly, the energy gap (*E*_g_) shown in Figure 3e decreases from 2.82 eV to 2.66 eV (Table 2). The obtained values are slightly lower than in previously reported data [34]. This most likely arises due to a more defective graphitic structure, which introduces a number of trap states that lower the band gap, thus making it more photoactive.

The reflectance spectra of g-C_3_N_4_ samples synthesized from melamine and cyanuric acid are shown in Figure 3c. The band gap energy (*E*_g_) decreases to some degree by the increase in the melamine mass ratio, equaling 2.65 eV (Table 2). The measured value agrees with the literature [37]. The same trend can be observed here as for the melamine-synthesized samples.

Table 2 presents the calculated values of the indirect band gap for the prepared g-C_3_N_4_. The band gap energy (*E*_g_) was calculated from the reflectance spectra using the Kubelka–Munk remission function and plotting the function against the photon energy. The band gaps were obtained by extrapolating the sharp linear absorption peak to the x-axis.

**Figure 4:**
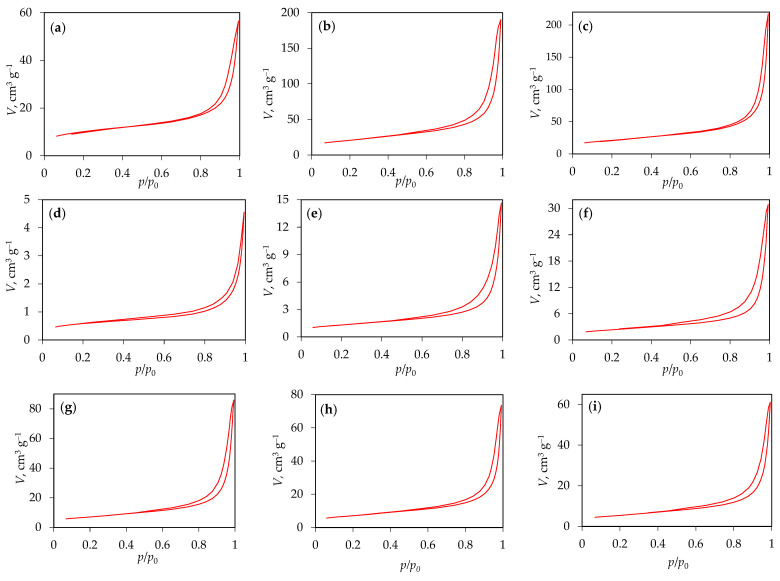
Nitrogen adsorption/desorption isotherms for g-C_3_N_4_ samples (**a**) g-CN-U-450, (**b**) g-CN-U-500, (**c**) g-CN-U-550, (**d**) g-CN-M-450, (**e**) g-CN-M-550, (**f**) g-CN-M-550, (**g**) g-CN-MCA-1:1, (**h**) g-CN-MCA-1:2, and (**i**) g-CN-MCA-2:1.

Figure 4a–I shows nitrogen adsorption/desorption isotherms of g-C_3_N_4_ samples synthesized from urea, melamine, and the melamine and cyanuric acid complex.

According to the updated IUPAC classification [38], there are eight types of adsorption isotherms, six of which were already identified in the 1985 IUPAC Manual on Reporting Physisorption Data for Gas/Solid Systems. The isotherms obtained are predominantly similar to type III, indicating relatively weak interactions between the adsorbent and the adsorbate. In type III isotherms, the adsorbed molecules tend to cluster around the most energetically favorable sites on the surface of a non-porous or icroporous solid. However, since the results show a hysteresis loop, which is atypical for reversible type III isotherms, it is more likely that the observed isotherms correspond to type IV(a). Type IV(a) isotherms are characteristic of mesoporous materials, where the presence of hysteresis is associated with capillary condensation and evaporation within the mesopores. (1) This phenomenon occurs when the pore width exceeds a critical threshold, which is typically the case for pores larger than ~4 nm [39,40,41], and this is the case for all the samples analyzed (Table 3).

**Figure 5:**
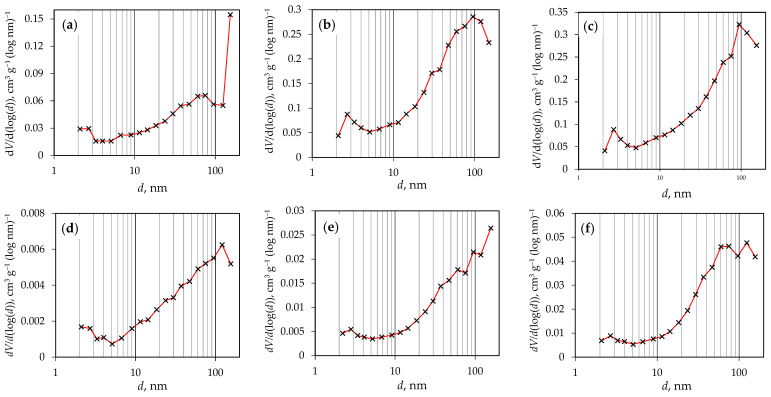

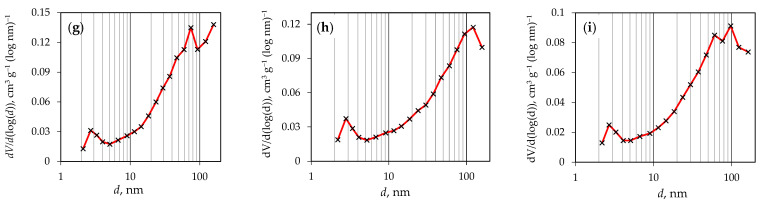
Pore size distribution of prepared g-C_3_N_4_ samples (**a**) g-CN-U-450, (**b**) g-CN-U-500, (**c**) g-CN-U-550, (**d**) g-CN-M-450, (**e**) g-CN-M-550, (**f**) g-CN-M-550, (**g**) g-CN-MCA-1:1, (**h**) g-CN-MCA-1:2, and (**i**) g-CN-MCA-2:1.

The shape of the adsorption hysteresis loop provides information about the size distribution, geometry, and connectivity of the pores. In all the samples, the observed H3 hysteresis loop is an indication of a pore network consisting of macropores that are not completely filled with pore condensate. This observation is consistent with the pore size distribution, which confirms the presence of macropores larger than 50 nm (Figure 5). Based on the pore size distribution obtained, all the samples can be classified as mesoporous to macroporous materials, with most pores falling in the 10–200 nm range. A small number of micropores was also observed, with the total volume of micropores being less than 0.01 cm^3^ g^−1^ for all the samples.

Table 3 shows the values for the specific surface area and the average pore diameter of the g-C_3_N_4_ samples.

The data show that the average pore diameter of the samples is between 8.0 and 17.5 nm, confirming that the g-C_3_N_4_ samples produced have a mesoporous structure. This also confirms the presence of pores larger than 4 nm, which would be the reason for the capillary condensation and evaporation, as well as the hysteresis observed in the isotherms. In addition, as the synthesis temperature increases, there is a significant increase in both the specific surface area and pore diameter for the samples prepared from urea and melamine, with the influence of urea, which produces the highest specific surface area, being the most favorable. In contrast, when melamine and cyanuric acid were combined, the specific surface area of the samples was larger than that of the melamine samples but smaller than that of the urea samples. Furthermore, no significant influence of the ratio of melamine and cyanuric acid on the specific surface area was found.

**Figure 6:**
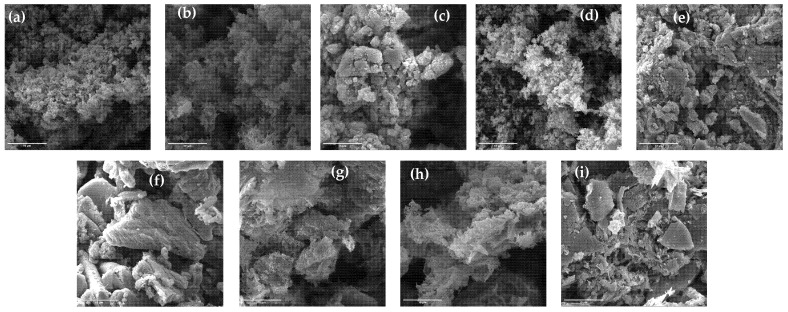
SEM images of the samples (**a**) g-CN-U-450, (**b**) g-CN-U-500, (**c**) g-CN-U-550, (**d**) g-CN-M-450, I g-CN-M-550, (**f**) g-CN-M-550, (**g**) g-CN-MCA-1:1, (**h**) g-CN-MCA-1:2, and (**i**) g-CN-MCA-2:1.

SEM images of the samples synthesized from urea can be seen in Figure 6a–c. A visible increase in the structure and pore sizes with the increase in synthesis temperature agrees with the values of specific surface area [31].

The morphology of melamine-derived g-C_3_N_4_ samples is given in Figure 6d–f. The g-CN-M-450 sample shows quite a disordered structure with a small number of pores. With the increase in processing temperature, the g-C_3_N_4_ structure becomes more layered with larger structural objects and prominent pores. This is proven by the increase in specific surface area and the pore size [43].

Figure 6g–i presents SEM images of g-C_3_N_4_ samples from the melamine and cyanuric acid complex. By doubling the amount of melamine compared to the cyanuric acid (2:1), randomly oriented agglomerated crystallites occur [37]. The samples synthesized at different temperatures exhibit distinct morphologies, which directly impact surface area, porosity, and active site accessibility—critical parameters for photocatalytic efficiency. For instance, the sample prepared at 500 °C shows a more porous and loosely stacked structure, which correlates with enhanced light absorption and improved charge separation, resulting in superior photocatalytic activity.

### 3.2. Adsorption, Photolysis, and Photocatalytic Degradation of Procaine

The photocatalytic activity of prepared g-C_3_N_4_ samples synthesized from different precursors (urea, melamine, and a complex of melamine and cyanuric acid) and different temperatures (450, 500, and 550 °C) was evaluated for the degradation of procaine (PRO) under visible (VIS), UV-A, and simulated solar light (SSL). The adsorption tests were carried out in the dark for 2 h as a separate experiment to determine the adsorption/desorption equilibrium, and the results showed that the equilibrium was reached at 30 min.

Prior to the adsorption and photocatalytic test, photolysis, i.e., the degradation of procaine under a light source, was carried out. The results showed insignificant degradation of procaine under all the light sources.

The adsorption of procaine for all the g-C_3_N_4_ samples was negligible (under 10%). Therefore, adsorption kinetics were not determined. From the photolytic and adsorption tests, it can be concluded that the degradation of procaine happens solely due to the light radiation on the samples.

**Figure 7:**
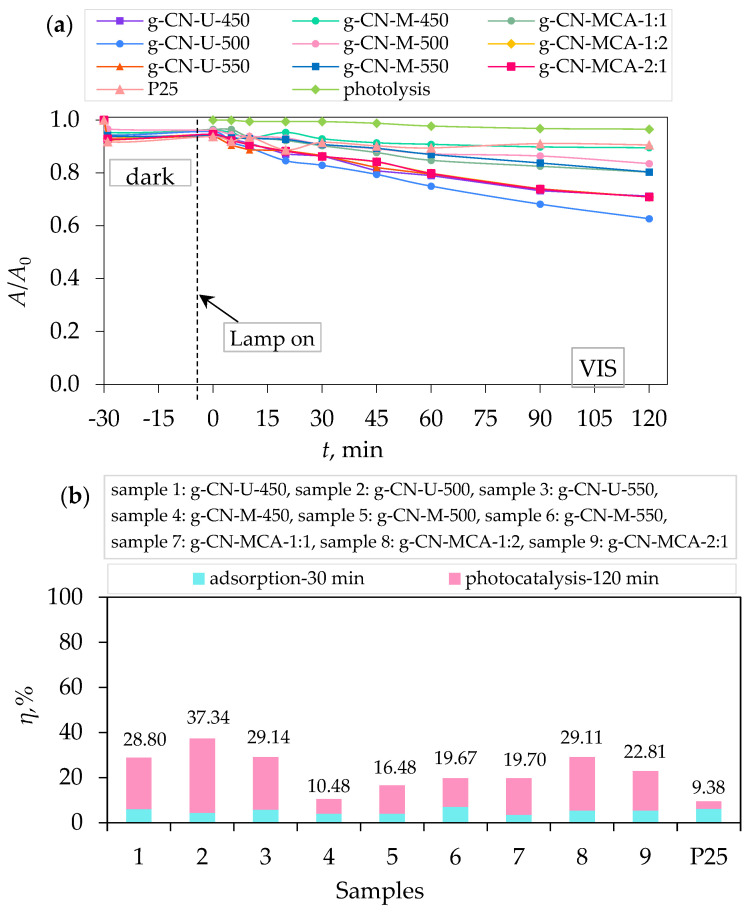
(**a**) Photocatalytic degradation of procaine by g-C_3_N_4_ samples, and (**b**) removal efficiency by synergistic effects of adsorption and photocatalysis under visible light.

**Figure 8:**
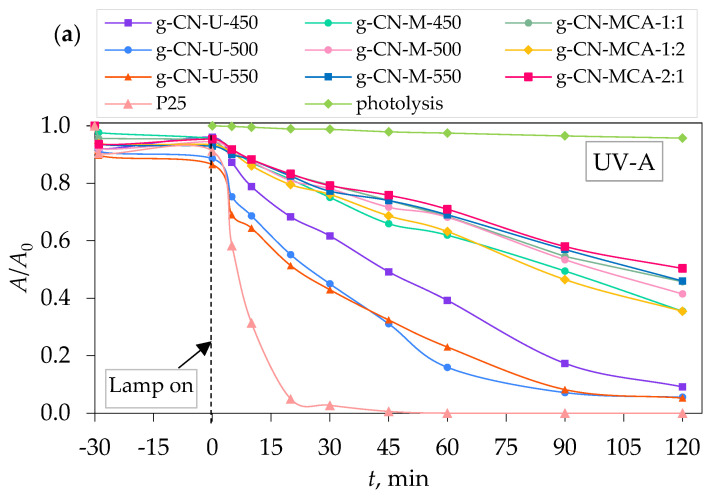

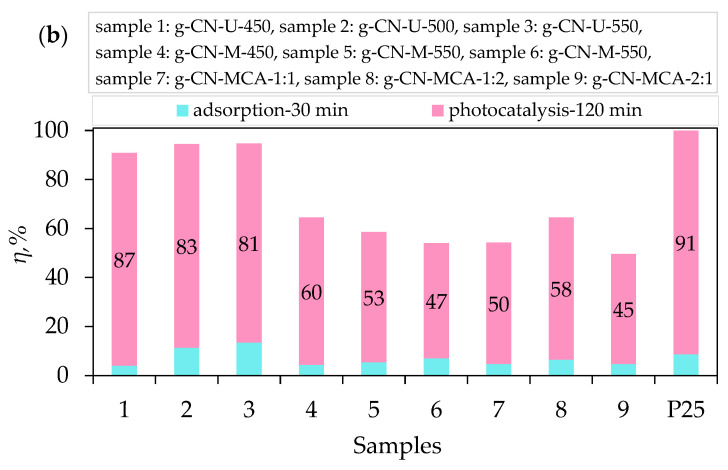
(**a**) Photocatalytic degradation of procaine by g-C_3_N_4_ samples, and (**b**) removal efficiency by synergistic effects of adsorption and photocatalysis under UV-A light.

**Figure 9:**
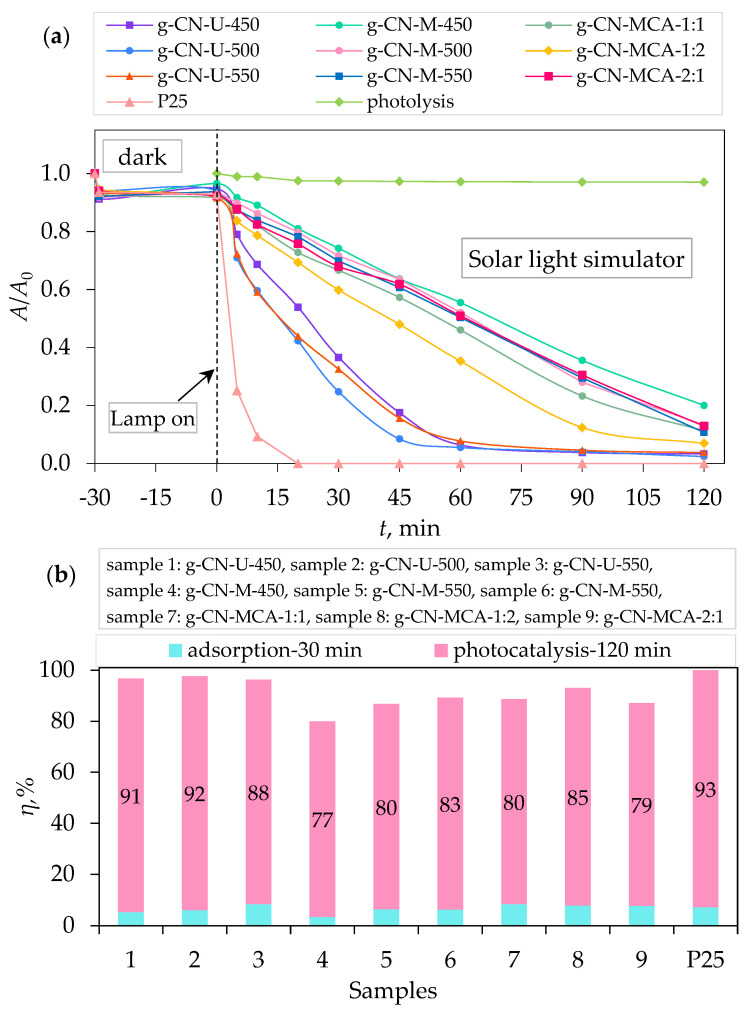
(**a**) Photocatalytic degradation of procaine by g-C_3_N_4_ samples, and (**b**) removal efficiency by synergistic effects of adsorption and photocatalysis under simulated solar light.

Figure 7a shows the photocatalytic activity of g-C_3_N_4_ samples under visible light. It can be seen that the urea-derived sample g-CN-U-500 exhibits the highest photocatalytic activity, with the degradation efficiency of PRO at 37.34% after 2 h irradiation (Figure 7b). In Figure 8a, samples g-CN-U-500 and g-CN-U-550 show the highest photocatalytic degradation of PRO under UV-A light after 2 h. The degradation efficiency of the samples was 94.48% and 94.72%, respectively (Figure 8b). Similar results can be achieved from photocatalytic activity under simulated solar light (Figure 9a). The highest degradation of PRO was exhibited by g-CN-U-500 at 97.57% after 2 h (Figure 9b). The urea-derived samples under simulated solar light mainly achieve their maximum degradation efficiency at 60 min. For the g-CN-U-500, the efficiency after 60 min equals 94.47%, which differs by only 3% from the result after 2 h. The obtained results are consistent with their surface area, meaning that the increase in specific surface area results in a higher procaine degradation rate.

In comparison to the commercially available TiO_2_ P25, all the prepared g-C_3_N_4_ samples exhibited a higher degradation efficiency under VIS light. To be specific, g-CN-U-500, as the sample with the best performance under visible light, had a four times better degradation efficiency than P25. Contrarily, the P25 samples showed a significantly higher degradation rate under UV-A and SSL, as can be concluded from the literature [42,44].

The kinetic constant (*k*) for the pseudo-first-order model was determined from the slope of the plot of ln(*C*/*C*_0_) against irradiation time (*t*):

(1) $$ln(C/C_{0})=kt$$

Table 4, Table 5 and Table 6 present the pseudo-first-order kinetic constant values (*k*_1_, min^−1^) and half-life times (*t*_1/2_), along with the corresponding determination coefficients (*R*^2^) for PRO removal using both the synthesized g-C_3_N_4_ samples and the commercial TiO_2_ P25. The high determination coefficients (*R*^2^ > 0.90) suggest that the degradation process of PRO adheres to the pseudo-first-order kinetic model.

By examining the kinetic constant values and half-life times, it is evident that the urea-derived samples degraded procaine notably faster than the samples synthesized with melamine or melamine and cyanuric acid complex.

### 3.3. Photocatalytic Mechanisms of g-C_3_N_4_

To determine degradation mechanisms, it is necessary to identify the role of reactive oxygen species (ROS) and surface charges (*h*^+^, *e*^−^) that affect the photocatalytic removal of PRO; therefore, scavenger/interfering species were used. The sample that exhibited the highest degradation efficiency (g-CN-U-500) was subjected to photocatalytic tests similar to the already performed ones, but with added scavengers under simulated solar light. SSL was chosen as a representative type of radiation because it consists of both UV-A and visible light.

**Figure 10:**
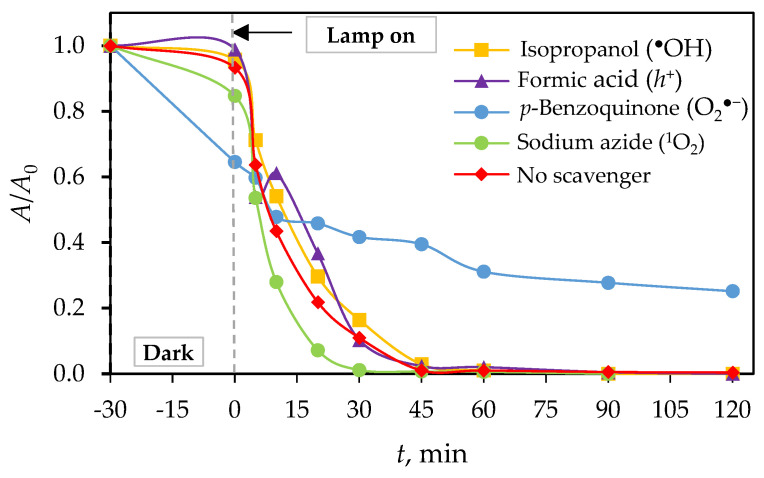
Photocatalytic degradation of procaine by g-C_3_N_4_ synthesized from urea at 500 °C, 2 h in the presence of different scavenger agents under simulated solar light.

The photocatalytic test for PRO removal by g-CN-U-500 under SSL is shown in Figure 10. Alcohols like isopropanol and tert-butanol are often used as quenchers of hydroxyl radicals, formic acid, or potassium iodide for holes, and sodium azide scavenges singlet oxygen [45,46,47]. Although photocatalytic removal of pharmaceuticals is most often mediated by hydroxyl radicals [48,49], adding the alcohol in the procaine solution did not cause any inhibition effect, i.e., the kinetic rate was similar to the reaction without the scavenger (Table 7). The reason for the suppressed generation of hydroxyl radicals may lie in the electrochemical performance of the applied substrate. The top of the valence band of the g-C_3_N_4_ photocatalyst is less positive than the redox potential of OH^−^/^•^OH [50,51]. Procaine also showed resistance to two other scavengers, eliminating the participation of *h*^+^ and ^1^O_2_ in its removal. The attached kinetics (Figure 10) for the scavenging test indicate a significant reduction in the analyte with the addition of *p*-benzoquinone and its synergistic activity of sorption and photocatalysis, where the half-time of removal increased from 8.3 to 91.2 min. Therefore, it was indicated that the main role in PRO degradation is attributed to the superoxide radicals (O_2_^•−^), possibly formed by direct electron transfer from the conduction band to dissolved oxygen in solution, where a scavenging agent can trap electrons and become reduced, itself [52,53]. These results are in agreement with a previously published study where photocatalysis was also performed on a g-C_3_N_4_-based photocatalyst [51].

Table 8 presents a comparison of the degradation efficiencies of various other g-C_3_N_4_ photocatalysts for the degradation of different organic pollutants in water. Although the organic pollutants vary, the degradation efficiency reported for the pharmaceutical sulfamethazine is comparable to that achieved with the g-C_3_N_4_ samples synthesized in this study.

Future investigations will explore the immobilization of the most active g-C_3_N_4_ photocatalyst, particularly the urea-derived sample synthesized at 500 °C, in the form of thin films or coatings on porous three-dimensional substrates to assess its reusability and stability. Such structured systems are expected to facilitate catalyst recovery, enhance mechanical robustness, and enable integration into continuous-flow photoreactors for practical wastewater treatment applications.

## 4. Conclusions

In this study, nanoparticles of graphitic carbon nitride (g-C_3_N_4_) were prepared and characterized. The synthesis was carried out via thermal polymerization using different precursors, including urea, melamine, and a complex of melamine and cyanuric acid in mass ratios of 1:1, 1:2, and 2:1. The urea and melamine were polymerized in a muffle furnace at three different temperatures (450 °C, 500 °C, and 550 °C), while the melamine–cyanuric acid complex was calcined at 550 °C only. Following synthesis, the materials were characterized using X-ray diffraction (XRD), diffuse reflectance spectroscopy (DRS), Fourier transform infrared spectroscopy (FTIR), and BET specific surface area analysis. Additionally, adsorption and photocatalytic tests were performed to evaluate the degradation of the local anesthetic drug procaine. The XRD analysis of urea-derived samples confirmed the formation of the g-C_3_N_4_ phase in all cases. In contrast, the XRD patterns of melamine-derived samples revealed the presence of melem as the dominant phase at 450 °C, while the g-C_3_N_4_ phase started forming at higher temperatures (550 °C). This indicated that the synthesis temperature influenced only the formation of g-C_3_N_4_ from melamine. DRS analysis showed that the urea-derived g-C_3_N_4_ samples had slightly higher band gap energies (2.81–2.88 eV) compared to the typical literature value (~2.7 eV), possibly due to structural defects. On the other hand, the band gap values of melamine-derived samples at 500 °C and 550 °C (2.72 eV and 2.66 eV, respectively), as well as those from the melamine–cyanuric acid complex (2.65–2.72 eV), were in agreement with the literature data. FTIR spectra confirmed the successful synthesis of graphitic carbon nitride, as evidenced by characteristic vibrational bands in all samples. BET analysis revealed that the urea-derived samples had higher specific surface areas compared to those synthesized from melamine or the melamine–cyanuric acid complex. All the samples were determined to be mesoporous based on their pore size distribution. Adsorption tests showed that all the g-C_3_N_4_ samples had minimal surface affinity toward procaine, with adsorption efficiencies below 10%. The scavenger experiments identified p-benzoquinone, a superoxide radical (O_2_•^−^) scavenger, as having the most significant influence on the photocatalytic degradation pathway. Ultimately, the sample synthesized from urea at 500 °C (g-CN-U-500) exhibited the highest photocatalytic removal efficiency of procaine, achieving 37.34% under visible light, 94.48% under UV-A, and 97.57% under simulated solar light irradiation after 2 h of exposure.

## Figures and Tables

**Table 1:** Synthesis temperature and synthesis time of g-C_3_N_4_ samples in a muffle furnace.

Precursor	Sample Name	*T*, °C	*t*, min
Urea	g-CN-U-450 (1)	450	120 min, 3 °C min^−1^
	g-CN-U-500 (2)	500	
	g-CN-U-550 (3)	550	
Melamine	g-CN-M-450 (4)	450	
	g-CN-M-500 (5)	500	
	g-CN-M-550 (6)	550	
Melamine + cyanuric acid 1:1	g-CN-MCA-1:1 (7)	550	
Melamine + cyanuric acid 1:2	g-CN-MCA-1:2 (8)	550	
Melamine + cyanuric acid 2:1	g-CN-MCA-2:1 (9)	550	

**Table 2:** The calculated values of the indirect band gap for the prepared g-C_3_N_4_.

g-C_3_N_4_ Sample	Indirect Band Gap (*E*_g_, eV)
g-CN-U-450	2.81
g-CN-U-500	2.81
g-CN-U-550	2.88
g-CN-M-450	2.82
g-CN-M-500	2.72
g-CN-M-550	2.66
g-CN-MCA-1:1	2.69
g-CN-MCA-1:2	2.72
g-CN-MCA-2:1	2.65

**Table 3:** The specific surface area values and average pore diameter of the prepared g-C_3_N_4_ samples.

g-C_3_N_4_ Sample	Specific Surface Area	Average Pore Diameter
	$S_{BET},\;m^{2}g^{-1}$	$d_{average},\;nm$
g-CN-U-450	36.0	8.0
g-CN-U-500	73.7	13.3
g-CN-U-550	74.2	13.0
g-CN-M-450	2.1	10.1
g-CN-M-500	4.7	14.4
g-CN-M-550	8.3	17.5
g-CN-MCA-1:1	24.8	16.4
g-CN-MCA-1:2	25.1	13.7
g-CN-MCA-2:1	19.1	16.2
P25 ^1^	48.1 [42]	13.7 [42]

^1^ Degussa (Evonik, Germany) P25, Aeroxide TiO_2_.

**Table 4:** The values of the pseudo-first-order kinetic constant (*k*_1_), half-life time (*t*_1/2_), and determination coefficient (*R*^2^) of procaine removal by g-C_3_N_4_ samples under visible light.

g-C_3_N_4_ Sample	*k*_1_, min^−1^	*t*_1/2_*,* min	*R* ^2^
g-CN-U-450	0.0027	256.72	0.9797
g-CN-U-500	0.0330	21.00	0.9930
g-CN-U-550	0.0024	288.81	0.9836
g-CN-M-450	0.0006	1155.25	0.7594
g-CN-M-500	0.0017	407.73	0.9735
g-CN-M-550	0.0011	630.13	0.9149
g-CN-MCA-1:1	0.0014	495.11	0.9908
g-CN-MCA-1:2	0.0026	266.60	0.9928
g-CN-MCA-2:1	0.0017	407.73	0.9289
P25	0.0002	3465.74	0.0631

**Table 5:** The values of the pseudo-first-order kinetic constant (*k*_1_), half-life time (*t*_1/2_), and determination coefficient (*R*^2^) of procaine removal by g-C_3_N_4_ samples under UV-A light.

g-C_3_N_4_ Sample	*k*_1_, min^−1^	*t*_1/2_, min	*R* ^2^
g-CN-U-450	0.0185	37.47	0.9645
g-CN-U-500	0.0291	23.82	0.9886
g-CN-U-550	0.0251	27.62	0.9774
g-CN-M-450	0.0071	97.63	0.9949
g-CN-M-500	0.0059	117.48	0.9807
g-CN-M-550	0.0052	133.30	0.9878
g-CN-MCA-1:1	0.0058	119.51	0.9862
g-CN-MCA-1:2	0.0074	93.67	0.9803
g-CN-MCA-2:1	0.0050	138.63	0.9813
P25	0.0002	5.48	0.9698

**Table 6:** The values of the pseudo-first-order kinetic constant (*k*_1_), half-life time (*t*_1/2_), and determination coefficient (*R*^2^) of procaine removal by g-C_3_N_4_ samples under simulated solar irradiation.

g-C_3_N_4_ Sample	*k*_1_, min^−1^	*t*_1/2_, min	*R* ^2^
g-CN-U-450	0.0396	17.50	0.9611
g-CN-U-500	0.0363	19.09	0.9006
g-CN-U-550	0.0341	20.33	0.9698
g-CN-M-450	0.0113	61.34	0.9858
g-CN-M-500	0.0137	50.59	0.9557
g-CN-M-550	0.0129	53.73	0.9741
g-CN-MCA-1:1	0.0153	45.30	0.9591
g-CN-MCA-1:2	0.0226	30.67	0.9498
g-CN-MCA-2:1	0.0121	57.28	0.9676
P25	0.2306	3.01	0.9942

**Table 7:** The values of kinetic constant, half-life time, and determination coefficient for scavenger studies.

Scavenger	*k*, min^−1^	*t*_1/2_, min	*R* ^2^
Isopropanol	0.0772	8.98	0.9850
Formic acid	0.0708	9.79	0.9506
*p*-benzoquinone	0.0076	91.20	0.9121
Sodium azide	0.0908	7.63	0.8986
No scavenger	0.0836	8.29	0.9558

**Table 8:** Comparison of degradation efficiencies of some g-C_3_N_4_ photocatalysts during the degradation of different organic pollutants in water.

Photocatalyst	Pollutant	Pollutant Concentration, mg L^−1^	Light Source	Degradation Efficiency, %	Reference
g-C_3_N_4_ (melamine)	diclofenac	3	Visible light	80	[54]
g-C_3_N_4_ (melamine)	RhB	10	Visible light	35	[55]
g-C_3_N_4_ (melamine)	RhB	-	Visible light	39.86	[56]
g-C_3_N_4_ (melamine)	phenol	10	Visible light	92.5	[57]
g-C_3_N_4_ (urea)	MB	3	Visible light	44	[58]
g-C_3_N_4_ (urea)	*p*-nitrophenol	10	Visible light	41	[59]
g-C_3_N_4_ (melamine- cyanuric acid 1:1)	sulfamethazine	10	Visible light	14	[30]
g-CN-U-450	PRO	10	Visible light	28.8	This work
g-CN-U-500				37.3	
g-CN-U-550				29.1	
g-CN-M-450				10.5	
g-CN-M-500				16.5	
g-CN-M-550				19.7	
g-CN-MCA-1:1				19.7	
g-CN-MCA-1:2				29.1	
g-CN-MCA-2:1				22.8	
P25				9.4	

## Data Availability

The original contributions presented in this study are included in the article/Supplementary Material. Further inquiries can be directed to the corresponding authors.

## Supplementary Materials

The following supporting information can be downloaded at: https://www.mdpi.com/article/10.3390/ma18112522/s1, Figure S1: Digital imaging of prepared photocatalyst (**a**) g-CN-U-450, (**b**) g-CN-U-500, (**c**) g-CN-U-550, (**d**) g-CN-M-450, (**e**) g-CN-M-500, (**f**) g-CN-M-550, (**g**) g-CN-MCA-1:1, (**h**) g-CN-MCA-1:2, (**i**) g-CN-MCA-2:1.
