# Supplementary material for: Influence of Different Precursors on Properties and Photocatalytic Activity of g-C3N4 Synthesized via Thermal Polymerization
Source: Materials (Basel). 2025 May 27;18(11):2522. doi: 10.3390/ma18112522 (PMC12156244; doi:10.3390/ma18112522)
Supplement: Supplementary file 1 [file materials-18-02522-s001.zip › materials-3632817-supplementary.pdf]

Influence of different precursors on properties and photocatalytic activity of g-C_3N_4 synthesized via thermal polymerization

Debora Briševac [1,*], Ivana Gabelica [1], Floren Radovanović-Perić [2], Kristina Tolić Čop [2], Gordana Matijašić [2], Davor Ljubas [1], Lidija Ćurković [1,*]

[1] Faculty of Mechanical Engineering and Naval Architecture, University of Zagreb, Ivana Lučića 5, 10000 Zagreb, Croatia; ivana.gabelica@fsb.unizg.hr (I.G.); davor.ljubas@fsb.unizg.hr (D.L.)

[2] Faculty of Chemical Engineering and Technology, University of Zagreb, Marulićev trg 19, 10000 Zagreb, Croatia; fradovano@fkit.unizg.hr (A.B.); ktolic@fkit.unizg.hr (K.T.Č.); gmatijas@fkit.unizg.hr (G.M.)

* Correspondence: debora.brisevac@fsb.unizg.hr (D.B.); lidija.curkovic@fsb.unizg.hr (L.Ć.)

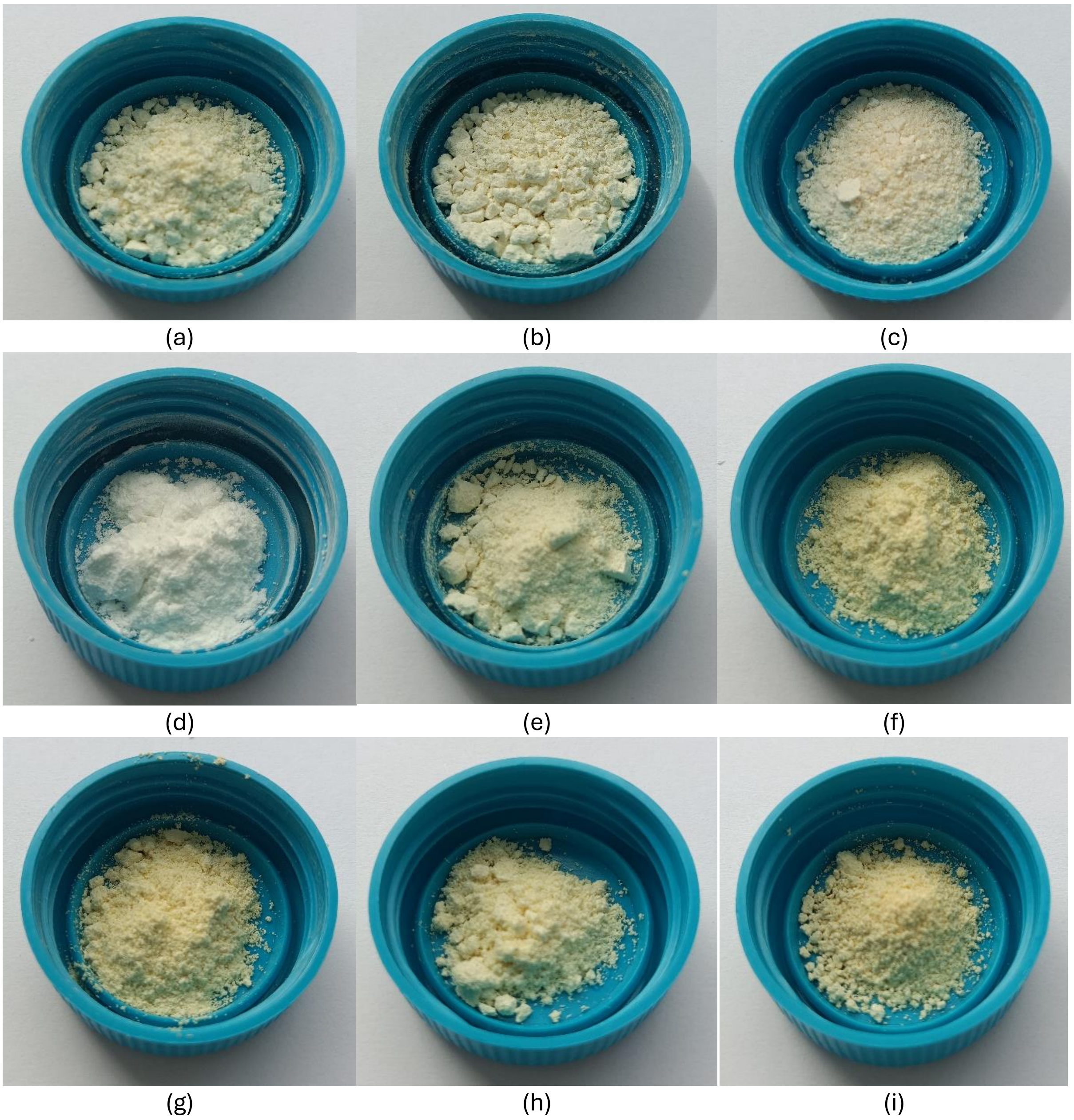

Figure S1. Digital imaging of prepared photocatalyst (**a**) g-CN-U-450, (**b**) g-CN-U-500, (**c**) g-CN-U-550, (**d**) g-CN-M-450, (**e**) g-CN-M-500, (**f**) g-CN-M-550, (**g**) g-CN-MCA-1:1, (**h**) g-CN-MCA-1:2 ,(**i**) g-CN-MCA-2:1.
